# Role of Glucagon and Its Receptor in the Pathogenesis of Diabetes

**DOI:** 10.3389/fendo.2022.928016

**Published:** 2022-06-16

**Authors:** Yunbo Jia, Yang Liu, Linlin Feng, Siyu Sun, Guangwei Sun

**Affiliations:** ^1^ Innovative Engineering Technology Research Center for Cell Therapy, Shengjing Hospital of China Medical University, Shenyang, China; ^2^ Department of Gastroenterology, Shengjing Hospital of China Medical University, Shenyang, China

**Keywords:** glucagon, diabetes, pathogenesis, glucagonocentric hypothesis, glucagon receptor, glucagon-like peptide 1

## Abstract

Various theories for the hormonal basis of diabetes have been proposed and debated over the past few decades. Insulin insufficiency was previously regarded as the only hormone deficiency directly leading to metabolic disorders associated with diabetes. Although glucagon and its receptor are ignored in this framework, an increasing number of studies have shown that they play essential roles in the development and progression of diabetes. However, the molecular mechanisms underlying the effects of glucagon are still not clear. In this review, recent research on the mechanisms by which glucagon and its receptor contribute to the pathogenesis of diabetes as well as correlations between *GCGR* mutation rates in populations and the occurrence of diabetes are summarized. Furthermore, we summarize how recent research clearly establishes glucagon as a potential therapeutic target for diabetes.

## 1 Introduction

Diabetes is a metabolic disorder characterized by hyperglycemia resulting from an absolute deficiency of insulin secretion (type 1 diabetes, T1D), or a combination of insulin resistance and an inadequate compensatory insulin secretion (type 2 diabetes, T2D) (1). However, each type of diabetes in animals and humans is accompanied by hyperglucagonemia (2–4), so glucagon excess is more critical to the development of diabetes than insulin deficiency (4, 5). Increasing evidence indicates that blocking glucagon and glucagon receptor (GCGR) can relieve hyperglycemia in animals and humans, clearly establishing the important roles of glucagon and GCGR in the pathogenesis of diabetes (6, 7).

Glucagon is a linear peptide containing 29 amino acids. It is secreted by islet α cells and mainly targets the liver cells (8). GCGR is a G-protein-coupled receptor (GPCR) mainly detected in islet β cells and liver cells (9). After glucagon specifically binds to GCGR, it promotes liver glycogen breakdown and increases blood glucose levels to stimulate insulin release (10, 11). Glucagon-like peptide 1 (GLP-1), mainly expressed in intestinal L cells, activates glucagon-like peptide-1 receptor (GLP-1R) to adjust metabolism (12, 13). Glucagon and GLP-1 are derived from the same biosynthetic precursor proglucagon and are involved in the regulation of lipid and cholic acid metabolism, thereby playing pivotal roles in glucose metabolism and the pathogenesis of diabetes (7, 12, 13).

In this review, we explore the controversial relationships between glucagon and metabolic disorders associated with diabetes based on recent research with an emphasis on recent evidence supporting the important role of glucagon. We also elucidate the correlation between *GCGR* mutations in populations and the occurrence of diabetes. Furthermore, we summarize drug strategies to provide a new basis for the treatment of diabetes.

## 2 Controversy Regarding the Role of Glucagon in Metabolic Disorders Associated With Diabetes

### 2.1 Insulinocentric Theory

The debate over the relative roles of hormones in the regulation of diabetes-related metabolic disorders has spanned decades. In 1921, the discovery of insulin was regarded as one of the greatest breakthroughs in the history of medicine. This led to the establishment of the insulinocentric view, which proposes that all diabetes-related metabolic disorders are directly caused by a lack of insulin secretion (14). Glucagon was not yet characterized and accordingly was not associated with these metabolic disorders. The insulinocentric theory was accepted for over half a century until Unger et al. proposed the bihormone theory at a conference in 1975 (15, 16).

### 2.2 Bihormonal Regulation

**Figure 1:**
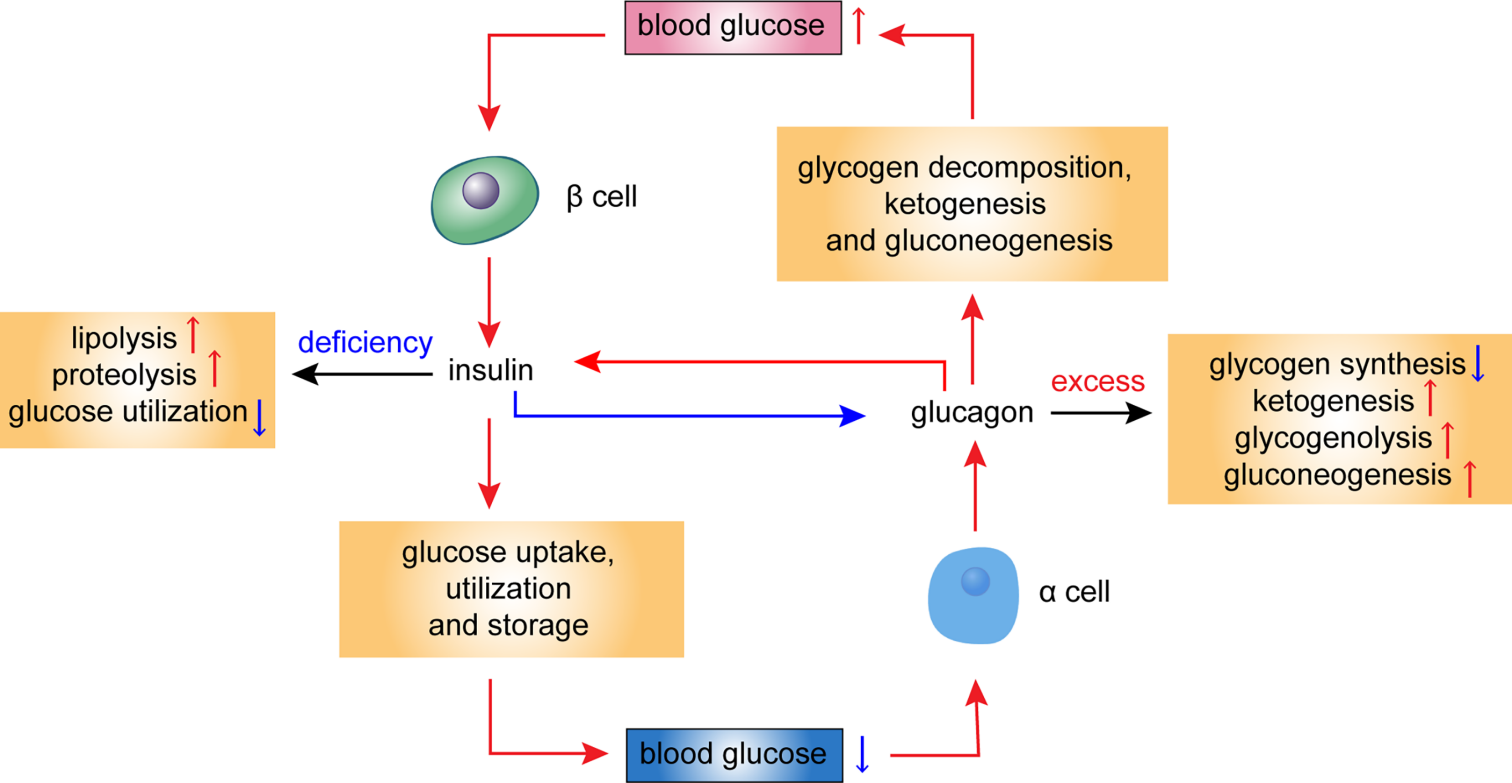
Hormonal regulation of glucose homeostasis in the islet cells. This diagram illustrates the metabolic effects of glucagon and insulin. Blood glucose levels influence secretion of insulin and glucagon. Insulin deficiency leads to elevated lipolysis, increased proteolysis, and decreased glucose utilization, while excess glucagon leads to decreased glycogen synthesis, increased ketogenesis, elevated glycogenolysis, and gluconeogenesis. Red arrows refer to a stimulatory effect, while blue arrows refer to an inhibitory effect.

According to the theory of bihormonal regulation, diabetes results from the abnormal secretion of both insulin and glucagon (15, 16). Some metabolic disorders associated with diabetes are directly caused by insulin deficiency, such as elevated lipolysis, increased proteolysis, and decreased glucose utilization. Others, such as decreased glycogen synthesis, increased ketogenesis, elevated hepatic glycogenolysis, and gluconeogenesis, are direct effects of excess glucagon (15–18) (**Figure 1**). Glucagon has glucogenic, ketogenic, and gluconeogenic functions and mediates severe endogenous hyperglycemia and hyperketonemia under a state of insulin deficiency; thus, it is a direct cause of the substantial increases in the levels of glucose and ketone in severe presentations of diabetes (19). In patients with diabetes with relatively steady levels of insulin, a rise in glucagon causes hyperglycemia and glycosuria (17). Glucagon suppression may be an effective adjunct to routine antihyperglycemic therapy in patients with diabetes (20–22).

### 2.3 Glucagonocentric Hypothesis

Glucagonocentric hypothesis was proposed by Unger et al. based on the following evidence: (a) hyperglucagonemia is present in all forms of diabetes; (b) marked hyperglucagonemia is caused by perfusing anti-insulin serum to the normal pancreas; (c) during a total insulin deficiency, all metabolic manifestations of diabetes can be suppressed by glucagon suppressors, like somatostatin, and in global *Gcgr* knockout (*Gcgr* ^-/-^) mice, demonstrating that β cell destruction does not cause diabetes (4). Thus, compared with insulin deficiency, glucagon excess plays a more essential role in the development of diabetes.

*Gcgr ^-/-^* mice were designed to further understand the role of GCGR in the development of diabetes; these mice do not respond to glucagon at any concentration, and their fasting blood glucose levels are lower than those of wild-type mice. These knockout mice exhibit enhanced glucose tolerance and elevated insulin sensitivity during insulin tolerance testing (23). When β cells of *Gcgr ^-/-^* mice were destroyed by streptozotocin (STZ) and insulin secretion was inhibited, animals did not develop hyperglycemia, suggesting that *Gcgr ^-/-^* mice do not develop T1D, even under a state of insulin deficiency (24). After the transient repair of defective *Gcgr* with an adenovirus vector, the blood glucose levels of the mice increased after β cell destruction (25). When *Gcgr* was inactivated again, blood glucose levels returned to normal, suggesting that in the absence of glucagon, insulin deficiency does not result in abnormal blood glucose levels, and that the abnormal blood glucose concentration caused by insulin deficiency can be restored by eliminating the effect of glucagon (25). Hence, blocking *Gcgr* can restore hyperglycemia in rodent models with insufficient insulin secretion; however, this effect requires a certain number of β cells (26). Active GLP-1 was identified in pancreatic perfusate from *Gcgr ^-/-^* but not wild-type mice (27), and FGF21 acts additively with GLP-1 to prevent insulinopenic diabetes in mice lacking glucagon action (28), which further reduces the risk of *Gcgr ^-/-^* mice developing diabetes. On the contrary, *Gcgr* knockout implies that glucagon cannot function normally, which can cause a series of metabolic problems, such as hyperglucagonemia and compensatory hyperplasia of α cells (23, 29, 30). Therefore, the above phenomena should be monitored in the development of GCGR antagonists. The therapeutic potential of GCGR is not fully recognized and should be a basis of further studies; however, the established animal models provide an effective means for the development of strategies to reduce the incidence of diabetes.

## 3 Mechanism by Which Glucagon Affects Insulin Secretion

In healthy people, high blood glucose stimulates β-cell insulin secretion, and glucagon secretion is suppressed; low blood glucose inhibits β-cell insulin secretion, and glucagon secretion is stimulated (**Figure 1**). Nevertheless, hyperglucagonemia was present in patients with diabetes, including T1D (31) and T2D (32). No significant difference of plasma glucagon level was found between T1D and T2D (31, 32). Absolute deficiency or relative deficiency of insulin secretion weakened the inhibition of insulin on glucagon (4).

Glucagon’s role in intra-islet paracrine regulation is essential. Svendsen et al. (27) used isolated perfused pancreas from wild-type, *Glp-1r* knockout, diphtheria toxin-induced proglucagon knockdown, β cell-specific *Gcgr* knockout, and *Gcgr^−/−^* mice to examine glucagon-induced insulin secretion. They found that paracrine glucagon actions are required for maintenance of normal insulin secretion, and intra-islet glucagon signaling involves the activation of both GCGR and GLP-1R. Loss of either GCGR or GLP-1R does not change insulin responses, whereas combined blockage of both receptors significantly reduces insulin secretion (27). Additionally, *Gcgr ^-/-^* mice show normal blood glucose levels and increased glucagon levels in glucose-stimulated insulin secretion (GSIS) tests after treatment with 10 mM (33) or 12 mM (27) glucose. This is similar to levels observed in control mice, suggesting that the insulin-promoting effect of glucagon is achieved mainly *via* GLP-1R. However, as the cognate downstream receptor of glucagon, the physiological significance of β-cell GCGR remains subtle. Zhang et al. (34) states that glucagon potentiates insulin secretion *via* β-cell GCGR at physiological but not high concentrations of glucose, and β-cell GCGR activation promotes GSIS more than GLP-1R in high fat diet. These findings indicate that GCGR contributes to glucose homeostasis maintenance during nutrient overload. These studies emphasized the indispensable roles of GCGR on β cells in mediating both the glucose balance and catabolic state and implied that GCGR is closely related to the pathogenesis of diabetes. Accordingly, studies of the mechanisms by which GCGR regulates insulin secretion are of great significance.

**Figure 2:**
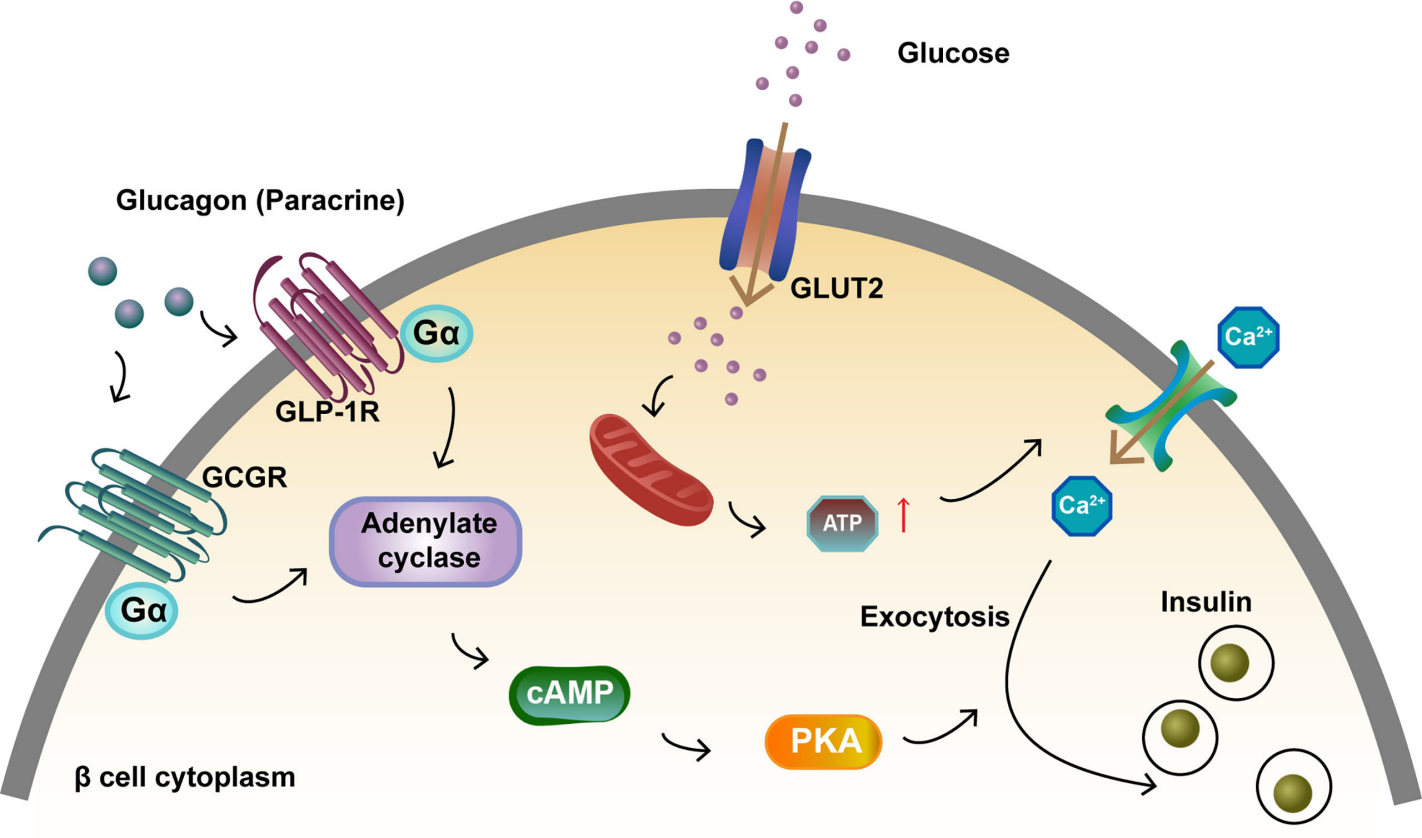
Activation of GCGR and GLP-1R to promote insulin secretion in islet β cells. Glucagon binds to GCGR and GLP-1R on β cells and the activated receptors engage the G protein Gαs. This results in adenylate cyclase activation and cAMP formation. Glucose binds to GLUT2, which increases ATP levels and intracellular calcium concentration, and enhances insulin exocytosis. The increase in intracellular cAMP levels activates PKA, which also promotes insulin exocytosis.

In pancreatic β cells, GLUT2, a glucose transporter protein, is required for GSIS (35). Glucose binding to GLUT2 is a key pathway leading to increased ATP levels, deionization, increased intracellular calcium concentration, and enhanced insulin exocytosis. GLUT1 expression decreased in *Gcgr^–/–^* mice but increased in wild-type mice after glucose stimulation (36). As a paracrine hormone, glucagon binds to GCGR with high affinity, while also exerting a “spillover” effect by binding to GLP-1R with low affinity (37). After glucagon binds to GCGR and GLP-1R on β cells, the activated receptors engage the G protein Gαs, which stimulate the generation of cyclic adenosine monophosphate (cAMP) (34, 38–40). The response of glucagon to glucose mainly depends on cAMP signaling in islet β cells and the increased cAMP level promotes insulin release (39, 41) (**Figure 2**).

## 4 Association of *GCGR* Mutations With Diabetes in Various Populations

T2D, also called non-insulin dependent diabetes mellitus, is a common disorder with complex traits. Multiple genomic scans have identified different loci associated with T2D, including a locus on chromosome 17q24-25 (42, 43) and *GCGR* on chromosome 17q25, which might be explained by linkage identified in the same region (44). GCGR mediates glucose homeostasis by binding to glucagon and may contribute to the pathogenesis of T2D and the development of β-cell dysfunction, resulting in a deficient insulin response in some patients with T2D. Further studies are needed to determine the effect of hepatic glucagon resistance on metabolic disorders and its association with the occurrence of diabetes. Chronic hyperglycemia increases the protein expression of GCGR in the liver and decreases downstream glucagon signaling, leading to liver glucagon resistance (45, 46). *GCGR* mutations may be related to hyperglucagonemia *via* the impairment of endogenous glucagon autofeedback, to high hepatic glucose output in T2D *via* elevated glycogenolysis and/or gluconeogenesis, and to abnormal insulin secretion *via* the glucagon resistance of β cells in T2D.

*GCGR* is regarded as a candidate gene for the pathogenesis of T2D and *GCGR* mutations with similar frequencies have been found associated with T2D (47). Polymorphisms in the *GCGR* gene are associated with T2D in Caucasians (48). The Gly40Ser variant of GCGR (c.118G >A) causes a change from glycine (at the 40th amino acid residue) to serine. In French and Sardinian familial T2D groups, 5% and 8% of randomly selected patients with diabetes, respectively, showed Gly40Ser mutations. These percentages are substantially higher than the frequencies of any other candidate gene mutations reported previously (47). Gough et al. examined patients from three geographically distinct regions in the United Kingdom and the Gly40Ser mutation was present in 15/691 patients with T2D and 1/425 geographically matched controls (48), suggesting that individuals with the Gly40Ser mutation may be predisposed to T2D. GCGR mutation frequencies have been examined in other populations and regions. However, the Gly40Ser mutation was not detected in studies involving subjects of Japanese (49–52), Finnish (53), Dutch (54), Utahans (55), German (54, 56), Russian (57), Indian Tamil (58), Han Chinese (59), Taiwanese (60), Brazilian (61), and Italian (44) descents. Another study (62) conducted in different areas of Sardinia did not find low insulin secretion in the population carrying this mutation in contrast to the earlier 1995 study (47). It showed that the Gly40Ser variation was not related to T2D in the Sardinian population and that its frequency varied among regions in Sardinia. Although no such association was found in Brazil, reduced insulin secretion was observed in Gly40Ser carriers (61). Based on a genetic analysis of 64 children with diabetes, the Gly40Ser mutation may be associated with T2D susceptibility in China (63). It reduces the binding of GCGR and glucagon and insulin secretion; this observation led Hansen to hypothesize that the Gly40Ser mutation in GCGR can lead to the abnormal functioning of islet β cells and may predispose carriers to diabetes, possibly by impairing glucagon-mediated signaling and decreasing the sensitivity of the target tissues to glucagon (64).

In addition to the relationship between the Gly40Ser mutation and T2D, an elevated frequency of *GCGR* mutations has been found in probands from multiple (affected sibling pair) families with T1D, also known as insulin-dependent diabetes; however, the lack of preferential transmission from heterozygous parents to affected siblings with T1D suggests population stratification (48). Overall, this Gly40Ser mutation may promote islet β-cell dysfunction, resulting in deficient insulin responses in patients with diabetes.

Together, these findings suggest that the contribution of *GCGR* to diabetes may vary and mutations in this gene play only a small role in determining the susceptibility of an individual to diabetes and the observed genetic heterogeneity of diabetes. Given the heterogeneity of the disease, the importance of *GCGR* for diabetes susceptibility may vary among ethnicities owing to the differences in genetic and environmental factors. *GCGR* is a polymorphic gene. The absence of a *GCGR* polymorphism (Gly40Ser) at one site does not rule out mutations associated with susceptibility to diabetes in other regions. For example, in addition to Gly40Ser, homozygous missense mutations (P86S) have been found in *GCGR*; these mutations contribute to the formation of an ineffective GCGR, resulting in hyperglycemia and extreme α-cell proliferation (65). Recent studies have reported 250 missense variants in human *GCGR* (66, 67). *GCGR* shows lower allelic diversity and fewer missense variants and variants with trait associations than the other class B1 GPCRs. These observations support the crucial role of the glucagon system in metabolism and indicate that the predominant signaling pathway mediating the physiological effects of GCGR is the one mediated by Gαs. These findings provide a clear link between molecular mechanisms and clinical phenotypes. The metabolic phenotypes related to several missense variants of *GCGR* have been investigated in case studies and in studies of genetically engineered animals, including V368M and V369M (68, 69). Further research is needed to explore the relationship between *GCGR* variants and diabetes.

## 5 Glucagon-Related Therapies for Diabetes

Several emerging glucagon-based therapies are under pre-clinical and clinical development.

### 5.1 GCGR Antagonism

GCGR antagonism has been proposed as a pharmacological approach to treat T1D or T2D, including the use of small molecule antagonists, monoclonal antibodies (mAb) against GCGR, and antisense oligonucleotides that reduce expression of the receptor (70–73). Relevant clinical trials have shown that they can reduce blood glucose levels through inhibition of glucagon action (74–76); however, several adverse effects, such as increased LDL-cholesterol (LDL-c), ALT level, and bodyweight, have been observed (74, 77).

#### 5.1.1 GCGR Antagonists

Several GCGR antagonists have been developed to improve glucose tolerance, insulin secretion, and glucose control in animals (78, 79), and have shown remarkable efficacy in patients with T2D, such as MK-0893, MK-3577, LY2409021 and LGD-6972 (76, 80–82). They upregulate circulating GLP-1 level by promoting intestinal L-cell proliferation and GLP-1 production in T2D (82). MK-0893 and MK-3577, which were advanced to phase II clinical trials, led to robust glucose lowering in patients with T2D; however, their adverse effects, such as increased LDL-c and ALT level, have hindered their clinical development (83–86). LY2409021 significantly reduced blood glucose and HbA1c levels with a lower risk of hypoglycemia (80, 81), but it increased liver fat (87). LGD-6972 is an allosteric GCGR antagonist, structurally different from other small molecule GCGR antagonists. It was well tolerated at all tested doses and did not cause hypoglycemia (88, 89), but additional details on biochemical differentiation are lacking and this compound does not appear to be under active clinical development (71).

#### 5.1.2 GCGR mAbs

With the cessation of clinical trials of GCGR antagonists and better understanding of the protein structure of GCGR, antibodies against GCGR have been developed. GCGR mAbs have good specificity, strong targeting, and are relatively easy to source. They can not only return blood glucose and HbA1c to normal levels when administered to mice with T1D not treated with insulin (73), as well as patients with T1D (90), but also show a strong hypoglycemic effect in mice and monkeys with T2D (91, 92). They can even induce β cell regeneration by the transdifferentiation of a portion of pancreatic α cells or δ cells into β cells (93). REMD 477 is a fully competitive mAb against GCGR. A single dose of REMD-477 significantly reduces insulin requirement in patients with T1D, which improves glycemic control in patients without serious adverse reactions (90). Another GCGR mAb, REGN1193, has good safety and tolerability, but transient elevation of transaminases was also observed (94). Overall, GCGR mAbs are promising for improving glycemic control and have great research promise.

#### 5.1.3 GCGR Antisense Oligonucleotides (GR-ASO)

GR-ASO inhibits the effect of glucagon mainly by decreasing the expression of GCGR mRNA (95). The intraperitoneal administration of GR-ASO to db/db mice and Zucker diabetic fatty (ZDF) rats decreases (nearly normalizes) non-fasting blood glucose levels (95). GR-ASO improves β-cell function (i.e., improves the insulin response to intraperitoneal glucose stimulation) and substantially improves glucose tolerance in normal and ZDF rats. However, *Gcgr* ^-/-^ mice and other animals treated with GR-ASO show extensive islet α-cell proliferation and significantly elevated circulating proglucagon-related peptide levels (96). Recently, ISIS-GCGRRx (76), IONIS-GCGRRxN (97), and ISIS 325568 (98) have been shown to attenuate glucagon-stimulated hepatic glucose production and glucose fluctuations. They support the treatment of GR-ASO in patients with T2D.

### 5.2 GLP-1R Agonists

The most well-characterized biological function of GLP-1 is to potentiate glucose-dependent insulin secretion, which makes the GLP-1R an attractive target in the treatment of T2D (99). Thus, GLP-1R agonists are clinically used as anti-diabetic drugs (100). Glucagon not only acts to antagonize insulin in the fasting state but also functions in the fed state and promotes insulin secretion to maintain normal blood glucose levels (34). The insulin-promoting properties of glucagon are mediated by GCGR and GLP-1R in β cells (27, 33, 101); however, GLP-1R is the main receptor to exert an insulin-stimulating effect (101). It is reasonable to assume that even with *GCGR* mutations in β cells, glucagon binding to GLP-1R exerts an insulin-promoting effect that can reduce blood glucose concentrations in patients with diabetes. Although GLP-1R agonists have been used for the treatment of diabetes, their efficacy is limited by target receptor desensitization and downregulation *via* the recruitment of β-arrestins (102, 103). GLP-1R agonists with decreased β-arrestin-2 recruitment have shown promising effects in recent preclinical and clinical studies (104). Understanding the mechanisms of action may resolve these issues with the application of GLP-1R agonists.

### 5.3 GCGR and GLP-1R Co-Agonists

Owing to the traditional view that the main effect of glucagon is to increase blood glucose levels, the idea of increasing glucagon concentration as a means of reducing glucose levels initially met resistance. Nevertheless, the action of glucagon on GCGR and GLP-1R (regulators of insulin secretion and energy metabolism) has a significant effect on systemic glucose homeostasis (105). On the one hand, GCGR and GLP-1R co-agonists can activate GLP-1R to promote insulin secretion and then reduce blood glucose. On the other hand, they can activate GCGR, promote lipid metabolism and reduce body weight (106–108). Since human islets have more mixed α-β cell interfaces, the ratio of GCGR to GLP-1R may be particularly vital to human islet function (8, 109). SAR425899 is a novel polypeptide with a co-excitatory effect on GCGR and GLP-1R, which can reduce blood glucose and HbA1c levels and reduce body weight in patients with T2D; however, it has an adverse effect on the gastrointestinal tract (110). It also improves postprandial blood glucose control by significantly enhancing β cell function and slowing glucose absorption rate (111). These findings highlight the possible clinical relevance of dual agonist peptides that simultaneously stimulate the synthesis of GCGR and GLP-1R and may drive the development of novel antidiabetic drugs.

## 6 Conclusions

In this review, we provide a clear overview of various theories of hormonal regulation of diabetes, with a focus on the essential roles of glucagon and its specific receptor in the pathogenesis of diabetes. Although GCGR and glucagon play important roles in diabetes, the mechanisms and role of mutations still needs to be explored. We summarized the pleiotropic effects of glucagon, future research prospects, and the development of novel therapeutic strategies. This area of research remains challenging but exciting. Further research on islet α cells, glucagon, and GCGR signaling pathways is expected to provide a basis for developing new strategies for diabetes prevention.

## Author Contributions

YJ wrote the manuscript. GS designed and critically reviewed the manuscript. SS critically revised the manuscript. YL and LF supervised the writing of the manuscript. All authors contributed to the article and approved the submitted version.

## Funding

Funding was received from 345 talent project of Shengjing hospital of China Medical University and the National Natural Science Foundation of China, grant # 82070683.

## Conflict of Interest

The authors declare that the research was conducted in the absence of any commercial or financial relationships that could be construed as a potential conflict of interest.

## Publisher’s Note

All claims expressed in this article are solely those of the authors and do not necessarily represent those of their affiliated organizations, or those of the publisher, the editors and the reviewers. Any product that may be evaluated in this article, or claim that may be made by its manufacturer, is not guaranteed or endorsed by the publisher.
